# New Insights Into c.815_824dup Pathogenic Variant of *BRCA1* in Inherited Breast Cancer: A Founder Mutation of West African Origin

**DOI:** 10.3389/fonc.2021.810060

**Published:** 2022-01-13

**Authors:** Jean Pascal Demba Diop, Andréa Régina Gnilane Sène, Yacouba Dia, Seydi Abdoul Ba, Serigne Saliou Mbacke, Cheikh Ameth Tidiane Ly, Pierre Diaga Sarr, Doudou Diouf, Sidy Ka, Babacar Mbengue, Serigne Modou Kane Gueye, Pape Saloum Diop, Maguette Sylla Niang, Papa Madieye Gueye, Philomene Lopez Sall, Ahmadou Dem, Aynina Cisse, Alioune Dieye, Rokhaya Ndiaye

**Affiliations:** ^1^ Division of Human Genetics, Faculty of Medicine, Pharmacy and Odontology, University Cheikh Anta Diop (UCAD), Dakar, Senegal; ^2^ Joliot Curie Institute, Aristide Le Dantec Hospital, Dakar, Senegal; ^3^ Immunology Unit, Faculty of Medicine, Pharmacy and Odontology, University Cheikh Anta Diop (UCAD), Dakar, Senegal; ^4^ Gynecology Unit, Aristide Le Dantec Hospital, Dakar, Senegal; ^5^ Unit of General Surgery, General Hospital Idrissa Pouye, Dakar, Senegal; ^6^ Laboratory of Pharmaceutical Biochemistry, Faculty of Medicine, Pharmacy and Odontology, University Cheikh Anta Diop (UCAD), Dakar, Senegal

**Keywords:** inherited breast cancer, *BRCA1* gene, pathogenic variant, founder mutation, Africa

## Abstract

Founder mutations have been reported in *BRCA1* and *BCRA2* in different ethnic groups with inherited breast cancer. Testing of targeted mutations in specific populations is important for cancer prevention in mutation carriers. In Sub-Saharan Africa, only a few studies have reported specific founder mutations in inherited breast cancer. The pathogenic variant c.815_824dup of *BRCA1* has been reported as the most frequent among African American populations with inherited breast cancer and was supposed to have a West African origin. Recent report from Senegal identified this variant in women with inherited breast cancer at the highest frequency ever reported. The variant was linked to a common haplotype confirming its founder effect in West Africa. In this article, we review the mutation history of c.815_824dup and discuss how it spread out of Africa through the transatlantic slave trade.

## Introduction

About 5%–10% of breast cancer cases are associated with inherited susceptibility. A subset of these are linked to germline pathogenic variants in *BRCA1* and *BRCA2* tumor suppressor genes (1). Women who have inherited the *BRCA1* or the *BRCA2* mutation are at greater risk of developing breast and/or ovarian cancer (2, 3). Studies in different populations have identified pathogenic variants stored in *BRCA* mutation consortia databases: CIMBA (Consortium of Investigators of Modifiers of BRCA1/2), ENIGMA (Evidence-Based Network for the Interpretation of Germline Mutant Alleles), BRCA Share (formerly the UMD-BRCA1 mutations database), and BRCA Exchange (https://brcaexchange.org). Some of these variants are at very high frequencies in specific ethnic groups, suggesting their founding effect. In Sub-Saharan Africa, a few studies have reported specific founder mutations (4, 5). Recently, we have reported a founder mutation, c.815_824dup, of the *BRCA1* gene in Senegalese women with inherited breast cancer (6). This mutation is a duplication of 10 nucleotides (c.815_824dupAGCCATGTGG, p.Thr276AfsX14) located in exon 11 of *BRCA1* according to the Human Genome Variation Society (HGVS) nomenclature and leads to a frameshift and a truncated protein. A previous study on African American women has reported the variant as a founder mutation of West African Origin (7).

## History of the c.815_824dup *BRCA1* Pathogenic Variant

Review from the literature has shown that c.815_824dup was first described in 1997 in a breast cancer patient from the Ivory Coast living in the US who had a familial history of breast, ovarian, and prostate cancers (8). In 1999, Mefford et al. identified the variant in four other families of African origin: one from the Bahamas and three in the US (South Carolina, Florida, and Washington, DC) (7). It was suggested that the variant may be an ancient West African founder mutation (7). Thereafter, the variant was reported in other African admixture populations with inherited breast cancer throughout the world (Bahamas, Peru, Mexico, France, Spain, and North America) (9–17). Literature search showed that the variant has been reported in 23 studies (**Table 1**). A review by Friebel et al. (31) revealed that the c.815_824dup pathogenic variant was detected in 76 individuals from the CIMBA database, 34 individuals from the Breast Cancer Information Core (BIC) database, and 16 individuals from ClinVar. Overall, the variant was identified in 155 patients. Of these, 100 were self-reported African ancestry (SRAA): 46 African American, 2 African (from Senegal), 6 Caribbean, and 46 of other African descent (31). The allelic frequency of the variant was estimated among African American women with inherited breast cancer at 16% in 2018 (15) and at 12% in 2020 (18).

**Table 1:** Studies reporting the c.815_824dup pathogenic variant throughout the world.

Region	Title	Authors	Year	Country	Number of patients with the mutation/Allelic frequency	Link to the article
**Africa**	Evidence for an ancient BRCA1 pathogenic variant in inherited breast cancer patients from Senegal	Ndiaye et al. (6)	2020	Senegal	27.7%	doi: 10.1038/s41525-020-0114-7
**USA**	Contribution of germline mutations in cancer predisposition genes to tumor etiology in young women diagnosed with invasive breast cancer	Palmer et al. (18)	2020	USA	12%	doi: 10.1093/jnci/djaa040
	Mutational spectrum in a worldwide study of 29,700 families with BRCA1 or BRCA2 mutations	Rebeck et al. (15)	2018	USA	16%	doi: 10.1002/humu.23406
	Contribution of Germline Predisposition Gene Mutations to Breast Cancer Risk in African American Women	Rummel et al. (19)	2017	USA	1	doi: 10.1007/s10549-017-4291-8
	A high frequency of BRCA mutations in young black women with breast cancer residing in Florida	Pal et al. (20)	2015	USA	2	doi: 10.1002/cncr.29645
	Deleterious BRCA1/2 mutations in an urban population of Black women	Lynce et al. (21)	2015	USA	1	doi: 10.1007/s10549-015-3527-8
	Inherited predisposition to breast cancer among African American women	Churpek et al. (22)	2014	USA	2	doi: 10.1007/s10549-014-3195-0
	Evaluation of BRCA1 mutations in an unselected patient population with triple-negative breast cancer	Rummel et al. (23)	2013	USA	1	doi: 10.1007/s10549-012-2348-2
	Recurrent BRCA1 and BRCA2 mutations in breast cancer patients of African ancestry.	Zhang et al. (24)	2012	USA	2	doi: 10.1007/s10549-012-2136-z
	Earlier age of onset of BRCA mutation-related cancers in subsequent generations.	Litton et al. (25)	2012	USA	1	doi: 10.1002/cncr.26284
	BRCA1 and BRCA2 mutations in women of different ethnicities undergoing testing for hereditary breast-ovarian cancer.	Hall et al. (26)	2009	USA	28	doi: 10.1002/cncr.24200
	Prevalence of pathogenic BRCA1 mutation carriers in 5 US racial/ethnic groups	John et al. (27)	2007	USA	1	doi: 10.1001/jama.298.24.2869
	Genetic testing in an ethnically diverse cohort of high-risk women: a comparative analysis of BRCA1 and BRCA2 mutations in American families of European and African ancestry	Nanda et al. (28)	2005	USA	1	doi: 10.1001/jama.294.15.1925
	BRCA1 and BRCA2 Mutations in a Study of African American breast cancer patients American Breast Cancer Patients	Pal et al. (29)	2004	USA	1	PMID: 15533909
	Breast cancer genetics in African Americans	Olopade et al. (16)	2003	USA	3	doi: 10.1002/cncr.11019
**Latino-America**	Evidence for a BRCA1 founder mutation in families of West African ancestry.	Mefford et al. (7)	1999	Bahamas US (South Carolina, Florida and Washington DC)	5	doi: 10.1086/302511
	Mutational analysis of BRCA1 and BRCA2 genes in Peruvian families with hereditary breast and ovarian cancer	Buleje et al. (30)	2017	Peru	1	doi: 10.1002/mgg3.301
	The prevalence of BRCA1 and BRCA2 mutations among young Mexican women with triple-negative breast cancer	Garza et al. (10)	2015	Mexico	5	doi: 10.1007/s10549-015-3312-8
	Prevalence of BRCA1 and BRCA2 mutations in unselected breast cancer patients from Peru	Abugattas et al. (9)	2015	Peru	1	doi: 10.1111/cge.12505
	The spectrum of BRCA1 and BRCA2 mutations in breast cancer patients in the Bahamas	Akbari et al. (14)	2014	Bahamas	3	doi: 10.1111/cge.12132
	A high prevalence of BRCA1 mutations among breast cancer patients from the Bahamas	Donenberg et al. (11)	2011	Bahamas	3	doi: 10.1007/s10549-010-1156-9
	Prevalence of BRCA mutations and founder effect in high-risk Hispanic families	Weitzel et al. (12)	2005	Mexico	1	doi: 10.1158/1055-9965.EPI-05-0072

## *BRCA1* c.815_824dup Pathogenic Variant in Africa

In Africa, until October 2021, only one study from our team has reported the variant in patients diagnosed with inherited breast cancer (6). The variant was identified in 15 index cases with a family history of breast and/or ovarian cancer, out of 27 recruited. Allelic frequency was estimated at 27.7% compared to sporadic breast cancer cases (5%) and healthy controls (0.55%) (6). We have then implemented in our laboratory a PCR genotyping method of the variant for oncogenetic counseling. Screening of the variant in 19 other index cases with a family history of breast cancer identified the variant in heterozygous state in 11 cases. The variant was also found in 8 out of 36 patients diagnosed without a known family history of breast cancer at under 50 years of age (allelic frequency estimated at 11.11%). These data strongly support the implication of the variant in inherited breast cancer.

Overall, the c.815_824dup variant was identified in 26 families out of 46 (allelic frequency estimated at 28.26%). Screening of the variant in healthy relatives also identified the variant in 13 out of 29, confirming its implication in disease etiology. This is the highest frequency of this variant ever reported in a population. The allelic frequency of c.815_824dup is rarely documented throughout the different BRCA variant databases. Available information is from gnomAD and TopMed, where frequencies were estimated at 2.09726 × 10^−5^ and 7.15273 × 10^−5^, respectively, worldwide and in the African population. Other databases revealed the presence of this variant in individuals with African origin [Allele Frequency Aggregator (ALFA), CIMBA, ClinVar, and BIC databases], while any allelic frequency was documented. Larger studies in African countries are needed to estimate the exact allelic frequency of the variant among the African population.

## West African Origin of *BRCA1* c.815_824dup

Haplotype analysis of seven microsatellite markers flanking the *BRCA1* gene and distributed in 2.15 Mb has identified a common haplotype of ~400 kb in Senegalese patients. This haplotype was not observed in any of the 48 healthy controls studied and is shorter than the one reported among African Americans spanning 700 kb (6). Age estimation of the Senegalese haplotype was 1,400 years, while it was 200 years in the US (7). Subsequently, we hypothesized that the variant appeared first in West Africa and spread throughout the world by population migration.

## Discussion

**Figure 1:**
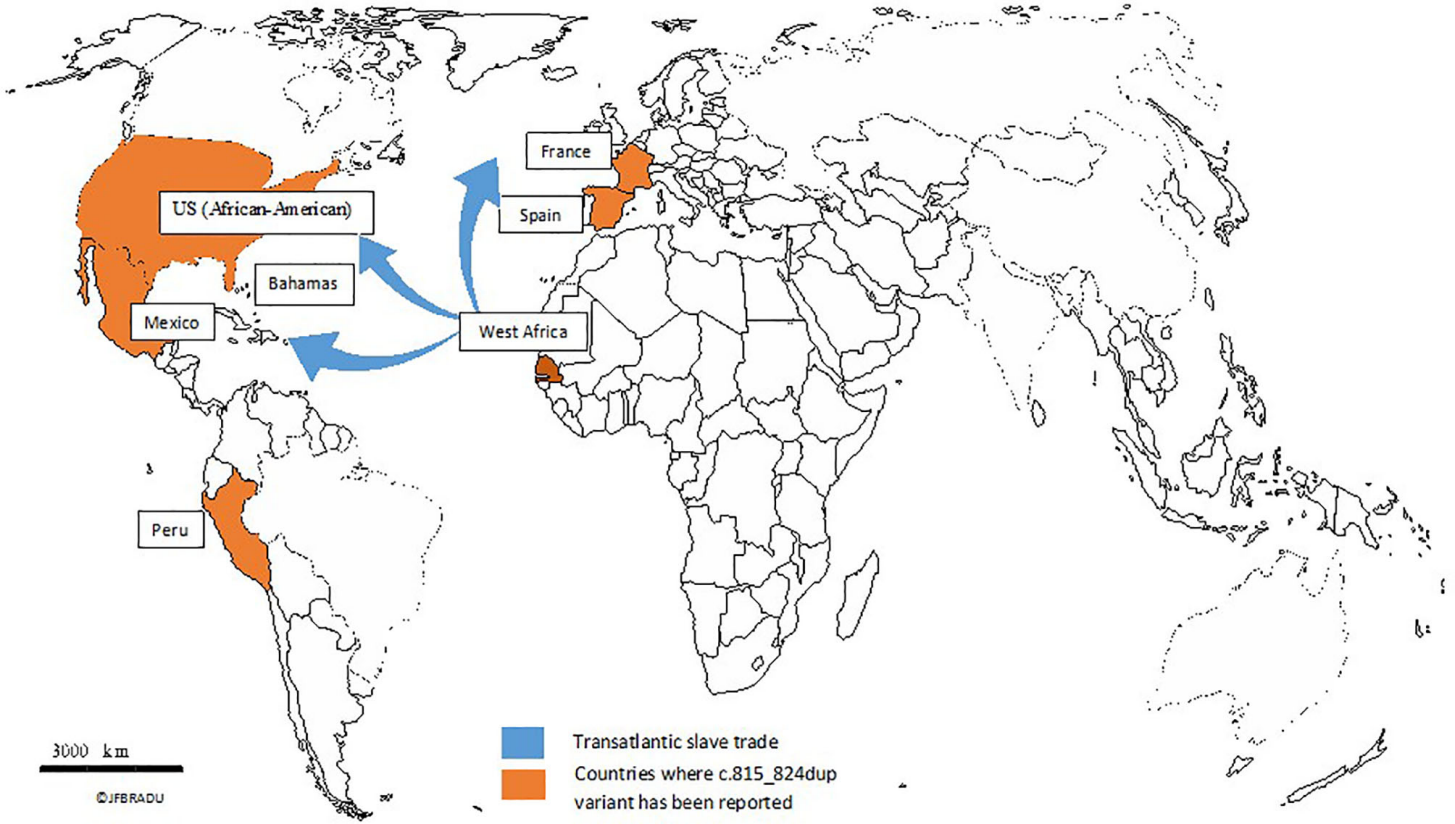
Distribution of the BRCA1 c.815_824dup variant through the transatlantic slave trade from West Africa to other continents.

Founder mutations linked to inherited breast cancer have been reported in *BRCA1* and *BRCA2* genes in different populations. The most known is the Ashkenazi Jewish pathogenic variant c.66_67AG (p.Glu23fs) reported in the US, Europe, Asia, and among Afrikaners from South Africa, while it was not reported in Sub-Saharan Africa, where other mutations have been identified (15, 26). In Northern Africa, a few founder mutations have been linked to inherited breast cancer: c.5309G>T (p.Gly1770Val) reported in five unrelated Moroccan families (32) and c.5335delC (p.Gln1779Asnfs) reported in Egypt (33). In Sub-Saharan Africa, the most frequent founder mutations linked to inherited breast cancer are c.303T>G (p.Tyr101Ter), reported in 4 out of 434 patients in Nigeria (5); c.2641G>T (p.Glu881Ter), identified in 5 patients out of 90 in South Africa (4); and c.815_824dup, identified in 26 out of 46 Senegalese families (6). The high allelic frequency of the c.815_824dup variant (28.26%) combined with the common haplotype observed in patients bearing the mutation suggested a founder effect in West Africa. Studies from Nigeria and Burkina Faso have not reported the variant in inherited breast cancer cases (5, 34). Then, it is actually of interest to screen the variant in other West African countries, particularly those located along the coast such as Ghana, Guinea-Bissau, Benin, Ivory Coast, Guinea, Togo, Sierra Leone, Liberia, and The Gambia. It would also be of interest to screen other neighboring countries to Senegal, such as Mali and Burkina Faso, where slaves have been deported toward West African coasts. From these countries, three played a key role in the transatlantic slave trade during the 18th century. The Senegal, Ghana, and Benin borders served as departure ports of slaves from Africa to the US, the Caribbean, and Europe. Since the distribution of the *BRCA1* c.815_824dup variant around the world overlapped black population migration, we have hypothesized that the variant first appeared in West Africa and spread out to the other continents (**Figure 1**). The high frequency of the variant among African Americans (18) and Hispanic people living in the Caribbean and Europe (13, 31), the African descent status of people harboring the variant through genomic databases, and the shorter single haplotype observed in Senegalese patients (6) all together support the African founding effect of c.815_824dup. The variant has spread out from Africa to other continents essentially through the transatlantic slave trade. Screening in other African countries may also highlight the implication of the transcontinental slave trade in the distribution of the variant across Africa.

*BRCA1* mutations have also been involved in the occurrence of other cancers, including prostate and pancreatic cancer. The c.815_824dup variant has been associated with a risk up to 16% in prostate cancer and 1% in pancreatic cancer at age 70 years, according to clinical genetic testing reports from the Myriad Genetics Laboratories. Results from our study also showed, in two index case carriers, male relatives diagnosed with pancreatic/prostate cancer. Hence, it will be of interest to screen the mutation in Senegalese patients with these cancers in order to evaluate the burden of this mutation in the Senegalese population.

Founder mutations are useful for disease prevention since genetic testing can be targeted. In low-income settings, this will allow more rapid and less expensive testing and enable risk assessment and medical follow-up of people at risk. Studies focusing on a cohort of individuals with the same mutation will also be of interest to identify factors affecting penetrance, phenotype variability, or environmental risk modifiers for a better follow-up of affected individuals.

## Author Contributions

JD, RN, AS, YD, SB, SM, CL, and PDS drafted the manuscript. DD, SK, BM, SG, PD, MN, PG, PLS, ADe, AC, and ADi gave advise on drafting the manuscript. All authors contributed to the article and approved the submitted version.

## Funding

This research project was funded by the Ministry of Higher Education and Research of the Republic of Senegal through the 2015 FIRST program.

## Conflict of Interest

The authors declare that the research was conducted in the absence of any commercial or financial relationships that could be construed as a potential conflict of interest.

## Publisher’s Note

All claims expressed in this article are solely those of the authors and do not necessarily represent those of their affiliated organizations, or those of the publisher, the editors and the reviewers. Any product that may be evaluated in this article, or claim that may be made by its manufacturer, is not guaranteed or endorsed by the publisher.
